# Identification of the awareness level by the public of Arab countries toward COVID-19: cross-sectional study following an outbreak

**DOI:** 10.1186/s40545-020-00247-x

**Published:** 2020-07-15

**Authors:** Ruwidah Bonyan, Aseel Fuad Al-Karasneh, Faris El-Dahiyat, Ammar Abdulrahman Jairoun

**Affiliations:** 1 Department of Clinical Pharmacy, Faculty of Pharmacy, Jordan University of Science and Technology, Irbid, Jordan; 2 Department of Pharmacy Practice, College of Clinical Pharmacy, Imam Abdulrahman Bin Faisal University, Dammam, Saudi Arabia; 3 College of Pharmacy, Al Ain University, P.O Box 64141, Al Ain, United Arab Emirates; 4 Health and Safety Department, Dubai Municipality, Dubai, United Arab Emirates

**Keywords:** Awareness, COVID-19, Prevention, Factors, Arab countries, Kingdom of Saudi Arabia, Jordan, Palestine, Qatar, United Arab Emirates, Egypt

## Abstract

**Background:**

The novel coronavirus disease 2019 (COVID-19) pandemic is a global challenge. Improving public awareness about preventive measures and disseminating appropriate information about COVID-19 has a critical role in containing the disease.

**Aim:**

To evaluate and determine the factors that may affect the level of awareness and responses toward COVID-19 in Arab countries. The study could be helpful in identifying where more public education about COVID-19 is needed.

**Method:**

This cross-sectional, online descriptive questionnaire-based study was conducted in February and March 2020. A total of 485 participants from Arabic-speaking countries (Jordan, United Arab Emirates, the Kingdom of Saudi Arabia, Qatar, Palestine, and Egypt) were asked to complete this Arabic-translated survey using social media platforms (Facebook and WhatsApp).

**Result:**

In general, there was a good level of awareness of the participants regarding COVID-19. Higher awareness scores were significantly correlated with older participants [odds ratio (OR) 1.019; 95% CI 1.012–1.026], those who attended awareness campaigns [OR 1.212; 95% CI 1.081–1.358], secondary school education holders [OR 1.740; 95% CI 1.096–2.763], higher education diploma holders [OR 2.090; 95% CI 1.297–3.368], university degree holders [OR 1.969; 95% CI 1.265–3.066], those who have post-graduate education [OR 2.206; 95% CI 1.393–3.493], and healthcare employees [OR 1.259; 95% CI 1.025–1.547].

**Conclusions:**

The COVID-19 pandemic is causing global panic; thus, awareness and practices of preventive measures of COVID-19 should be increased through public educational campaigns, which should be planned in accordance with communities’ and countries’ attitudes toward COVID-19. Collaborative efforts between ministries of heath and residents of every country should be implemented.

## Introduction

For many decades, the emergence of new virus strains has been one of the challenging global concerns. For example, the world has recently faced the emergence of pandemic H1N1 in 2009, severe acute respiratory syndrome (SARS) in 2003, and Ebola virus disease in 2014–2016 [1].

In 2019, a novel strain of coronavirus, severe acute respiratory syndrome coronavirus 2 (SARS-CoV-2), which is responsible for mild to severe respiratory infections, emerged for the first time in a Chinese city called Wuhan [2]. This novel strain in the coronavirus family, which also includes the viruses that are responsible for both SARS and Middle East respiratory syndrome (MERS), causes coronavirus disease 2019 (COVID-19). These diseases share the symptoms of respiratory infection, such as cough and fever [3]. However, the number of cases of COVID-19 is higher than that of SARS or MERS; correspondingly, the number of deaths caused by COVID-19 is also higher [3–6]. As the outbreak of COVID-19 expanded beyond China, the World Health Organization (WHO) characterized it on March 11, 2020 as a pandemic disease [7].

Restrictive preventive measures and policies are needed to control the COVID-19 outbreak. Increasing people’s awareness is one potential measure to limit the spread of infectious disease that will have an impact on the economic, social, and mortality burdens of any infectious disease [8]. Once an infectious disease appears and expands in a country, the country’s center for disease control and prevention will attempt to control the expansion of the disease [9]. One of the methods used is quickly providing people with the necessary preventive knowledge to avoid the infection [10]. As soon as people are aware of the disease and preventive measures, they are able to change their behaviors and take careful measures to reduce their susceptibilities. Such measures can include frequent hand washing, avoiding direct contact with infected patients, vaccination (if available), and wearing masks. It has been shown that the administration of measles, rubella, and mumps vaccines are correlated with local measles outbreaks [11].

Changing people’s behaviors and responses during disease outbreaks can reduce the size of the outbreak rather than eliminate the presence of the disease itself. However, it is not fully understood to what extent individual behavioral responses can help to contain the disease [12]. Spreading awareness and health messages for contagious disease prevention could be achieved using various mechanisms, including through the media, public talks, and lectures. For example, spreading preventive knowledge through television was effective to control H1N1 influenza in the Mississippi Gulf Coast [13]. In addition, awareness campaigns, websites, and television were used for public education about SARS in Singapore [14].

Global travel is an important factor that influences both the risk of infectious disease spread or emergence, especially in the presence of worldwide reachable and fast transportation networks [15, 16]. Additionally, global economic growth and tourism are critical factors in increasing the volume of human mobility which can be obviously noticed in the high-income countries [15].

This study evaluates public awareness of COVID-19 in some Arab Gulf countries (KSA, UAE, and Qatar) where the volume of travel and mobility is very high due to its excellent economy and the availability of huge number of work vacancies. In return, other Arab countries were included in this study (Jordan, Palestine, and Egypt) that many of its citizens go to the Gulf countries in search of work. Hence, this study deals with different Arab countries in the same region which have high volume of human mobility between them. Furthermore, this study will highlight the impact of the non-citizens on the awareness level of the countries in this study.

The aim of the current study is to evaluate the level of awareness toward COVID-19 in Arab countries, as well as determine factors that affect the level of COVID-19 awareness among participants. Moreover, it will provide correlations between specific demographic characteristics and awareness of COVID-19, with the aim of helping to contain the COVID-19 pandemic. It will also compare the level of awareness among Arab countries.

## Methods and materials

### Study settings and design

This study consisted of an online descriptive cross-sectional questionnaire, with responses collected in February and March 2020. During this period, 485 participants from Arabic-speaking countries, including Egypt, Jordan, United Arab Emirates (UAE), the Kingdom of Saudi Arabia (KSA), Qatar, and Palestine, were invited via the social media platforms Facebook and WhatsApp to complete a validated web-based questionnaire.

### Study population (inclusion and exclusion criteria)

The population evaluated consisted of all contacted individuals aged 18 years old and above who were willing to participate in the study. Only those who spoke Arabic and were from Arabic-speaking countries, including Egypt, Jordan, UAE, KSA, Qatar, and Palestine, were included in the study. Contacted individuals who were less than 18 years old, did not speak Arabic, and/or did not wish to participate were excluded from the study.

### Sample size and sampling technique

The main question on which the sample size calculation was based was “What is the level of awareness about COVID-19 signs, symptoms, and preventive measures among the public?” In reviewing the literature, we did not find any data or results indicating what percentages of the population were aware of these topics. Thus, we estimated the proportion of the public who are aware of COVID-19 signs, symptoms, and preventive measures to be approximately 50%. The alpha level was set to 5% so that there was a 95% confidence level. The precision of the 95% confidence interval (CI) was fixed at 5% so that the width of the 95% CI was a maximum of 10%. According to the above assumptions, we needed a minimum sample of 385 participants. To recruit participants, a purposive sampling technique without a pre-determined sampling frame, in which the authors invited all of their social networking contacts as the data source to complete the questionnaire and share it with their own contacts, was used.

### Research instrument development

The research team developed the survey using the frequently asked questions displayed on the WHO website and what did they thought it is important to clarify about COVID-19. The items were translated from English into Arabic. A translated version then underwent review and assessment by subject experts to ensure the validity of design, appropriateness of content, comprehension, and reliability of the questionnaire. The content validity ratio (CVR) was then computed for all questionnaire items. Items with a CVR score of 0.78 or more were considered acceptable, while four items that had a CVR of less than 0.78 were deleted [17]. Next, the content validity index (CVI) was calculated to be 0.82, which indicated acceptable content validity of the entire questionnaire.

The validated version of the questionnaire was then piloted with 20 subjects to ensure relevance and clarity. Responses from the pilot stage were not included in the final study results. Construct validity was then assessed by performing exploratory factor analysis. Principal component analysis, followed by Varimax rotation with Kaiser-Meyer-Olkin (KMO) and Bartlett’s test of sphericity was conducted to determine the number of factors. Eigenvalues of 1 and items loaded of at least 0.40, with no cross-loading of items above 0.40 were satisfy the criteria of construct validity [18]. Finally, the reliability of the questionnaire was assessed by computing Cronbach’s α, for which a value of 0.78 was obtained, indicating acceptable internal consistency.

### Research instrument sections

The study instrument assessed awareness of the participants by asking questions about the sources of transmission, treatment, and vaccination of COVID-19. The questionnaire was divided into two parts. The first part included demographic data of the participants (age, gender, region of residence, education, employment, and health status), participants’ sources of information on COVID-19, and their health status on a scale of 0 to 10. The second part included COVID-19 awareness scale which assessed the participants’ awareness on preventive measures, treatment, vaccination, and source of transmission of COVID-19. The COVID-19 awareness scale utilized a five-point Likert scale (strongly agree, agree, neutral, disagree, and strongly disagree) for each of the 18 items.

### Ethical issues

The study was approved by Al Ain University Research Ethics Committee. Participation in the study was voluntary and the purpose of the study was explained in the cover page of the survey. If a participant continued to the next page, this was considered to be their consent to participate in the study. Participants’ identities were not recorded and confidentiality was assured.

### Statistical analysis

Data analysis was performed using SPSS version 24. Frequencies and percentages were used to display the qualitative variables, while mean ± standard deviation (SD) was used to summarize the quantitative variables. Then, the sum of awareness scores was calculated, ranging from a minimum sum of 18 to a maximum sum of 90 for each participant. Each participant’s score was calculated as the percentage and higher scores reflect better awareness. Kruskal–Wallis and Mann–Whitney *U* tests were used to determine the differences in awareness scores. Univariate and multiple logistic regression analyses were used to investigate the factors that are associated with awareness toward COVID-19. A *p* value < 0.05 indicated statistical significance.

## Results

### Demographic and baseline characteristics

The demographic and baseline information of the participants is displayed in Table 1. A total of 485 subjects participated in this study. The respondents were predominantly female (*n* = 392 or 80.8%). The average health status of the participants was 8.6 ± 1.8 (out of 10). The average age of the participants was 32.4 ± 10.4 years. Among the participants, 67 participants (13.2%) were aged 18–22, 67 participants (13.8%) were aged 23–26, 120 participants (24.7%) were aged 27–30, 105 participants (21.6%) were aged 31–36, 39 participants (8%) were aged 37–40, and 90 participants (18.6%) were aged ≥ 41. The countries of residence reported were as follows: 164 (33.8%) from Jordan, 21 (4.3%) from Palestine, 56 (11.5%) from Qatar, 128 (26.4%) from UAE, 87 (17.9%) from KSA, 9 (1.9%) from Egypt, and 20 (4.1%) from other countries. Of the 485 total participants, 233 (48%) reported the country they lived in. The education of the participants also varied. One percent (*n* = 5) were primary or elementary education holders, 11.1% (*n* = 54) were high secondary education holders, 8% (*n* = 39) were diploma holders, 60.4% (*n* = 293) were university degree holders, and 19.4% (*n* = 94) had attained post-graduate education. Among the total participants, 17.3% (*n* = 84) were students, 27.2% (*n* = 132) were unemployed, 22.9% (*n* = 111) were employees in the health sector, and 32.6% (*n* = 158) were employees in non-health sectors. Of the 111 participants who were employed in the health sector, 10 (9.0%) were physicians, 40 (36.0%) were nurses, 48 (43.2%) were pharmacists, and 13 (11.7%) were from other branches of the health sector. The respondents sought information about the novel coronavirus from different sources. Of the total, 370 (76.3%) obtained information about COVID-19 from social media, 174 (35.9%) from television, 190 (39.2%) from awareness campaigns, and 128 (26.4%) from family/friends. Among the total participants, 50.9% (*n* = 247) were interested in receiving more information about COVID-19.

**Table 1:** Demographic characteristics of study participants

Demographic	Groups	Frequency	Percentage
**Gender**	Male	93	19.2%
	Female	392	80.8%
**Age**	18–22	64	13.2%
	23–26	67	13.8%
	27–30	120	24.7%
	31-36	105	21.6%
	37–40	39	8.0%
	≥ 41	90	18.6%
**Country of residence**	Jordan	164	33.8%
	Palestine	21	4.3%
	Qatar	56	11.5%
	UAE	128	26.4%
	KSA	87	17.9%
	Egypt	9	1.9%
	Other	20	4.1%
**Having the same nationality as the country you live in**	Yes	233	48.0%
	No	252	52.0%
**Education**	Primary school/elementary	5	1%
	Secondary education	54	11.1%
	Diploma	39	8.0%
	University degree	293	60.4%
	Post-graduate degree	94	19.4%
**Employment**	Student	84	17.3%
	Unemployed	132	27.2%
	Employee in health sector	111	22.9%
	Employee in non-health sector	158	32.6%
**Health profession (*n*= 111)**	Physician	10	9.0%
	Nurse	40	36.0%
	Pharmacist	48	43.2%
	Other	13	11.7%
**Source of information**	Social media	370	76.3%
	TV	174	35.9%
	Awareness campaigns	190	39.2%
	Family/friends	128	26.4%
**Are you interested in receiving more information about COVID-19?**	Yes	247	50.9%
	No	238	49.1%

### COVID-19 awareness scale (construct validity)

Principle component analysis (PCA) extracted 3 factors with eigenvalues greater than 1 which explained 59.117% of the total variance. The Kaiser-Meyer-Olkin (KMO) value is 0.788 and Bartlett’s test of sphericity reached statistical significance (*p* < 0.001) which supported the factorability of the correlation matrix. Factor loadings between the 18 items of COVID-19 awareness scale and the 3 factors are displayed in Table 2. Factor 1 evaluating the awareness toward transmission and prevention measure of COVID-19 and comprised 11 items. Factor 2 assesses awareness toward vaccination and treatment of COVID-19 and comprised 4 items. Factor 3 assesses the cause and origin of COVID-19 and comprised 4 items.

**Table 2:** Exploratory factor analysis of the 18-item awareness scale toward COVID-19

Items	Factors		
	1	2	3
Isolation and quarantine of infected people are effective for preventing the spread of COVID-19.	**0.662**	0.154	0.199
Reporting symptoms of COVID-19 to local health authorities is important to prevent further disease transmission.	**0.631**	0.099	0.218
Frequent hand washing before preparing and/or eating foods is essential to prevent further COVID-19 transmission.	**0.620**	− 0.107	− 0.307
Hand washing after contact with possible contaminated materials or surfaces is important to prevent further COVID-19 transmission.	**0.619**	− 0.116	− 0.292
Consultation of a healthcare provider when you have a cough or fever or difficulty in breathing is critical for preventing COVID-19 spreading.	**0.595**	− 0.100	− 0.168
Travel bans to/from the areas of the disease should be implemented by the government to prevent COVID-19 spreading.	**0.572**	0.111	0.262
Traveling to China is considered as a risk of novel coronavirus infection.	**0.547**	0.057	0.321
Avoiding contact with sick people can prevent spread of disease.	**0.543**	0.193	0.159
Isolation of COVID-19 patients is important to achieve effective adoption of infection control measures.	**0.482**	0.218	0.168
Avoiding close contact with person who has an active respiratory symptom is essential to prevent further transmission of the disease.	**0.440**	0.121	0.219
Hand washing with soap and water for at least 20 s can prevent COVID-19 transmission.	**0.432**	− 0.007	0.068
Antibiotics are effective in COVID-19 treatment.	0.063	**0.811**	− 0.128
COVID-19 can be treated with the available antiviral medications.	0.016	**0.728**	− 0.035
Vaccination of COVID-19 is available as a protective measure.	0.095	**0.697**	− 0.112
There is no treatment for COVID-19 until now.	0.108	**0.466**	0.203
COVID-19 spread could be prevented by avoiding live animal contact.	0.257	− 0.026	**0.674**
The main source of novel coronavirus is animal.	0.066	0.063	**0.577**
Chinese goods are considered as a source of exposure to novel coronavirus.	0.021	− 0.262	**0.401**

Extraction method, principal component analysis; rotation method, Varimax with Kaiser normalization

### Assessment of awareness toward COVID-19

The average awareness score was 78.5% with a 95% confidence interval (CI) of 77.2–79.8. In general, the overall level of awareness of COVID-19 causes and prevention was good. Table 3 presents the distribution of awareness score according to demographic and baseline factors. Statistically significant associations were observed between COVID-19 awareness score and age groups (< 0.001), education (0.003), and employment (< 0.001). Older participants, those who attained a higher educational level and employees in the healthcare sector (< 0.001), were more likely to score higher in COVID-19 awareness score which tested awareness of COVID-19 causes and prevention (Table 3).

**Table 3:** COVID-19 awareness score according to demographic variables

Awareness items	Strongly agree		Agree		Neutral		Disagree		Strongly disagree	
	F	%	F	%	F	%	F	%	F	%
Isolation and quarantine of infected people are effective for preventing the spread of COVID-19.	374	77.1	94	19.4	14	2.9	3	0.6	0	0
Reporting symptoms of COVID-19 to local health authorities is important to prevent further disease transmission.	394	81.2	71	14.6	17	3.5	3	0.6	0	0
Frequent hand washing before preparing and/or eating foods is essential to prevent further COVID-19 transmission.	354	73.0	86	17.7	30	6.2	10	2.1	5	1.0
Hand washing after contact with possible contaminated materials or surfaces is important to prevent further COVID-19 transmission.	332	68.5	101	20.8	40	8.2	10	2.1	2	0.4
Consultation of a healthcare provider when you have a cough or fever or difficulty in breathing is critical for preventing COVID-19 spreading.	251	51.8	125	25.8	68	14.0	26	5.4	15	3.1
Travel bans to/from the areas of the disease should be implemented by the government to prevent COVID-19 spreading.	387	79.8	76	15.7	18	3.7	4	0.8	0	0
Traveling to China is considered as a risk of novel coronavirus infection.	339	69.9	122	25.2	22	4.5	2	0.4	0	0
Avoiding contact with sick people can prevent spread of disease.	284	58.6	159	32.8	29	6.0	11	2.3	2	0.4
Isolation of COVID-19 patients is important to achieve effective adoption of infection control measures.	442	91.1	35	7.2	8	1.6	0	0	0	0
Avoiding close contact with person who has an active respiratory symptom is essential to prevent further transmission of the disease.	252	52.0	143	29.5	56	11.5	21	4.3	13	2.7
Hand washing with soap and water for at least 20 s can prevent COVID-19 transmission.	194	40.0	228	47.0	46	9.5	15	3.1	2	0.4
Antibiotics are effective in COVID-19 treatment.	11	2.3	70	14.4	146	30.1	89	18.4	169	34.8
COVID-19 can be treated with the available antiviral medications.	18	3.7	60	12.4	205	42.3	138	28.5	64	13.2
Vaccination of COVID-19 is available as a protective measure.	15	3.1	58	12.0	157	32.4	112	23.1	143	29.5
There is no treatment for COVID-19 until now.	234	48.2	132	27.2	57	11.8	52	10.7	10	2.1
COVID-19 spread could be prevented by avoiding live animal contact.	134	27.6	226	46.6	82	16.9	82	16.9	5	1.0
The main source of novel coronavirus is animal.	202	41.6	138	28.5	96	19.8	35	7.2	14	2.9
Chinese goods are considered as a source of exposure to novel coronavirus.	101	20.8	128	26.4	89	18.4	102	21.0	65	13.4

*F* Frequency, *%* Percentage

The results of each question related to awareness toward COVID-19 causes and prevention are shown in Table 4.

**Table 4:** Participants’ awareness toward COVID-19

	COVID-19 awareness score			
Demographic variables	Mean ± SD		Median	***p*** value
**Gender**				
Male	**79.45**	**14.04**	**83.33**	**0.478**
Female	**78.27**	**14.43**	**77.78**	
**Age groups**				
18–22	**70.31**	**14.79**	**72.22**	**< 0.001**
23–26	**72.86**	**17.45**	**77.77**	
27–30	**74.54**	**14.22**	**77.78**	
31–36	**79.21**	**14.72**	**83.33**	
37–40	**80.11**	**13.45**	**83.33**	
≥ 41	**80.19**	**14.79**	**83.56**	
**Country of residence**				
Jordan	**79.40**	**14.52**	**83.33**	**0.199**
Palestine	**76.19**	**16.77**	**83.33**	
Qatar	**80.06**	**10.31**	**83.33**	
UAE	**78.99**	**16.24**	**80.56**	
KSA	**75.67**	**13.08**	**77.78**	
Egypt	**76.54**	**4.63**	**77.78**	
Other	**79.17**	**15.07**	**80.55**	
**Having the same nationality as the country you live in**				
Yes	**77.64**	**14.52**	**77.78**	**0.202**
No	**79.29**	**14.18**	**83.33**	
**Education**				
Primary school/elementary	**61.11**	**21.15**	**66.66**	**0.003**
Secondary school	**74.18**	**13.14**	**77.77**	
Diploma	**80.19**	**12.98**	**83.33**	
University degree	**78.54**	**15.17**	**77.78**	
Post-graduate degree	**81.08**	**11.29**	**83.33**	
**Employment**				**< 0.001**
Student	**73.81**	**13.79**	**77.77**	
Unemployed	**79.21**	**13.68**	**77.78**	
Employee in health sector	**83.13**	**12.17**	**83.33**	
Employee in non-health sector	**77.14**	**15.66**	**77.77**	
**Health profession**				
Physician	**83.33**	**9.44**	**80.50**	**0.446**
Nurse	**85.42**	**11.78**	**83.33**	
Pharmacist	**82.06**	**13.86**	**83.33**	
Other	**79.91**	**7.36**	**77.74**	

*p* values less than 0.05 were considered statistically significant, *p* values obtained from the Kruskal–Wallis and Mann–Whitney *U* tests

### Factors influencing awareness toward COVID-19 causes and prevention

Table 5 shows the results of univariate and multivariate logistic regression analyses for the factors associated with awareness of COVID-19.

**Table 5:** Univariate and multivariate analysis of factors associated with COVID-19 awareness score

Factors	COVID-19 awareness score							
	Univariate				Multivariate			
	OR	95% CI		*p* value	OR	95% CI		*p* value
Male	**1.073**	**0.941**	**1.224**	**0.292**	-------	-------	-------	-------
Age	**1.018**	**1.013**	**1.023**	**< 0.001**	**1.019**	**1.012**	**1.026**	**< 0.001**
Health status	**1.006**	**0.988**	**1.025**	**0.056**	-------	-------	-------	-------
Same nationality as country of residence	**0.906**	**0.818**	**1.004**	**0.059**	-------	-------	-------	-------
Awareness campaign	**1.327**	**1.192**	**1.477**	**< 0.001**	**1.212**	**1.081**	**1.358**	**0.001**
**Education (Ref. primary school/elementary)**								
Secondary school	**1.828**	**1.169**	**2.860**	**0.008**	**1.740**	**1.096**	**2.763**	**0.019**
Diploma	**2.578**	**1.623**	**4.094**	**< 0.001**	**2.090**	**1.297**	**3.368**	**0.002**
University degree	**2.328**	**1.516**	**3.575**	**< 0.001**	**1.969**	**1.265**	**3.066**	**0.003**
Post-graduate degree	**2.728**	**1.756**	**4.240**	**< 0.001**	**2.206**	**1.393**	**3.493**	**0.001**
**Country of residence (Ref. Jordan)**								
Palestine	**0.830**	**0.645**	**1.069**	**0.149**	-------	-------	-------	-------
Qatar	**1.041**	**0.871**	**1.245**	**0.656**	-------	-------	-------	-------
UAE	**0.975**	**0.853**	**1.115**	**0.716**	-------	-------	-------	-------
KSA	**0.807**	**0.697**	**0.933**	**0.004**	**0.751**	**0.617**	**0.913**	**0.004**
Egypt	**0.846**	**0.582**	**1.231**	**0.382**	-------	-------	-------	-------
Other	**0.986**	**0.753**	**1.291**	**0.916**	-------	-------	-------	-------
**Employment (Ref. student)**								
Unemployed	**1.352**	**1.162**	**1.573**	**< 0.001**	-------	-------	-------	-------
Employee in health sector	**1.749**	**1.485**	**2.060**	**< 0.001**	**1.259**	**1.025**	**1.547**	**0.028**
Employee in non-health sector	**1.198**	**1.037**	**1.384**	**0.014**	-------	-------	-------	-------

*p* values less than 0.05 were considered statistically significant, “---” not included in the multivariate logistic regression model

*OR* Odds ratio, *CI* Confidence interval

From the multivariate analysis, better awareness scores regarding COVID-19 were significantly associated with older participants [odds ratio (OR) 1.019; 95% CI 1.012–1.026], those who attended awareness campaigns [OR 1.212; 95% CI 1.081–1.358], secondary school education holders [OR 1.740; 95% CI 1.096–2.763], higher education diploma holders [OR 2.090; 95% CI 1.297–3.368], university degree holders [OR 1.969; 95% CI 1.265–3.066], those who pursued post-graduate education [OR 2.206; 95% CI 1.393–3.493], and employees in the healthcare sector [OR 1.259; 95% CI 1.025–1.547].

On the other hand, lower awareness scores toward COVID-19 were significantly associated with the participants residing in KSA [OR 0.751; 95% CI 0.617–0.913]. For more details, see Table 5.

## Discussion

This study was conducted after almost 2 months of COVID-19 outbreaks in China and Italy, when the entire world had heard about it and started preparing to contain the virus. At the same time, the data were collected before characterizing COVID-19 as pandemic by the WHO. This study aimed to determine whether people in Arab countries were aware of COVID-19 and what factors could influence people’s perceptions of this disease.

Data for this study were collected from different Arab countries (Jordan, Palestine, Qatar, KSA, UAE, and Egypt), with different sample sizes from each country. Therefore, our findings may not accurately reflect each nation’s perception toward COVID-19. In addition, knowledge of and behavior toward any infectious disease can be influenced by the severity of the illness and the prevalence of the disease [19, 12]. Furthermore, the awareness regarding a disease can change with time and with increases in global awareness [14]. In the current study, there were a high percentage of young participants, perhaps due to the method of contacting potential participants (via Facebook and WhatsApp). This could also have influenced the percentage of people who obtained information via social media.

In this study, three types of validity tests were measured (i.e., construct, content, and face validity). Notes provided by the participants in the pilot testing proving that the questionnaire items were straightforward, easy to understand, and in a reasonable order. For the content validity, CVI scale achieved a score of 0.82, which considered acceptable, indicating that the content of the questionnaire is well adopted into the Arabic environment. The exploratory factor analysis supported the three-factor structure of the questionnaire, which means that the awareness can be separated into three dimensions (transmission and prevention, vaccination and treatment, causes and origin of COVID-19).

Once the validity tests were finalized, the final version of the questionnaire was examined to measure its reliability, alpha value was 0.78, and this value reflects an acceptable internal consistency. Test retest was not measured in this study due to the difficulty in repeating the questionnaire on the same sample.

The present study found that the majority of the participants were aware of the ongoing COVID-19 outbreaks. The overall awareness regarding disease signs and symptoms, potential sources of infection, and prevention recommendations were deemed good. Similar findings were obtained in many other studies conducted in the past 5 years that also examined the awareness during outbreaks of other infectious diseases, including influenza H1N1, SARS, and avian influenza [20, 21].

From our demographic data, our participants were primarily in a good health status (average 8.6/10), young (average 32.4 years), and had at least a university degree (79%). The main source of information about COVID-19 was social media (76.3%), followed by awareness campaigns (39.2%) and television (35.9%). This is a logical finding because social media has become a feature of everyday life due to the widespread use of the Internet and smartphones. This was similar to the finding of a previous study that found that knowledge about MERS (another disease caused by a coronavirus) was mainly obtained from social media [22]. However, using social media as the main source of information may adversely affect individuals’ approaches toward preventive behaviors for the infectious diseases because the information shared in social media is provided by non-specialized people and is not scientifically reviewed [23].

Approximately half of the participants are interested in receiving more information about COVID-19 and this reflects their willingness to learn and the probability to change their behaviors to adopt more preventive measures. In contrast, the other half of the participants are not interested in receiving more information about COVID-19 which could be explained by the presence of almost 50% employed participants whom may do not have enough time to read or receive more information. In addition, at the time of collecting the data, a few cases were diagnosed with the infection in the Arab countries. So, the participants may not feel threatened by COVID-19.

Our study showed a high level of practicing preventive measures among participants. More than 80% of the participants avoided traveling to areas with outbreaks, avoided contact with animals or sick people, and washed their hands regularly.

The participants also showed a high level of awareness regarding the required precautionary procedures that should be taken on the governmental level during outbreaks. More than 95% of the participants agreed that governments should isolate the infected persons and restrict travel to and from the areas with outbreaks to avoid spreading COVID-19.

This study showed some misconceptions among participants. More than 10% thought that antibiotics and antiviral medications can treat COVID-19, and 46% thought that Chinese goods are a source of exposure to SARS-CoV-2. At the time of writing this article, there is no proven antiviral medication or vaccine for treatment or prevention of COVID-19. Thus, more information should be offered to the public regarding the availability of vaccines and effectiveness of treatments for COVID-19.

Regression analysis identified the factors influencing awareness and practices toward COVID-19. The results indicate that awareness scores were higher in older participants, those who attended awareness campaigns, those who have earned at least a secondary school certificate, and employees in the healthcare sector. On the other hand, awareness scores were lower for participants from KSA. This finding should encourage the ministry of health of KSA to conduct more awareness campaigns, as these campaigns were more reliable sources of information than social media. Older participants, compared to young participants, usually have more fear of the disease, and high levels of fear may be associated with increased practicing of preventive measures. In contrast, the younger participants may be less worried about the disease and accordingly demonstrate poor prevention behaviors [24]. This study also found that people with higher educational levels and those who are healthcare workers have more awareness and have a higher rate of practicing preventive behaviors. Furthermore, healthcare workers are more prone to contracting the infection than non-healthcare workers due to their close contact with infected individuals; accordingly, they are highly aware of COVID-19 causes and prevention strategies. This result is consistent with another study conducted to measure the awareness regarding MERS among healthcare workers [22]. In contrast, another study showed poor awareness and practice among healthcare worker toward MERS; the same study also finds that the poor awareness leads to poor prevention practice such as the lack of proper hygiene practicing [25].

Governments play a crucial role in crisis control via adoption of different preventive and protective policies. Increasing public awareness is one of such policies to control the disease spread. Publishing educational materials (videos, brochures, flyer, blogs, articles...) in different languages by using different platforms (TV, short message system, official websites...) and conducting awareness campaigns are examples of methods that can be implemented by the governments to achieve its goal in increasing the awareness level among the public. In view of that, the findings of this study recommend the governments to adopt more policies that would focus on the population with lower educational level and younger age to improve their awareness level.

Interestingly, between the involved countries in this study, there was no difference on the awareness level toward COVID-19 between citizens and non-citizens. Accordingly, travels between countries remained as the main factor that should be considered in combatting COVID-19 spread.

The COVID-19 pandemic has had a significant negative impact on many aspects of life around the world which requires more effort to improve the public response toward this disaster. Similar to other disasters, the COVID-19 pandemic could generate anxiety, fear, and depression between the public [26]. Thus, continuously providing the public with important response strategies and information about this pandemic could reduce the symptoms of anxiety and improve the level of public preparedness toward this pandemic [26, 27]. In contrast, long exposure to news about the pandemic-related events will be associated with more stress symptoms [28]. Therefore, the media and governments should focus on providing peoples with the appropriate information about the disease and practical response strategies rather than the pandemic-related events which will induce psychological influences and shock. Moreover, government or concerned authorities should develop a channel where the public can freely access and learned about the COVID-19 issues. In this way, we can assure the validity and accuracy of the information that public received about COVID-19 and reduce the stressful, uncontrolled, and confused information from the multimedia.

## Conclusions

The COVID-19 pandemic has had global negative impacts on social, economic, political, and health statuses of affected countries. To lessen the negative effects of this pandemic, further comprehensive improvement in public awareness about COVID-19 should be considered. Improving public awareness should be spearheaded by reliable health and governmental sectors. Social media, awareness campaigns, and television are very helpful platforms that should be adopted by decision makers in each country to raise community awareness and preventive attitudes toward COVID-19. Interestingly, the collaboration between the ministry of health and residents of every country plays a critical role in containing the COVID-19 pandemic. Because ministries of health tend to take action as outbreaks worsen, we expect a further improvement in the level of awareness as the number of COVID-19 cases announced in each country increases.

## Data Availability

All data will be provided upon request.
